# Environmental Toxicology and Human Health

**DOI:** 10.3390/ijms25010555

**Published:** 2023-12-31

**Authors:** Esref Demir, Sam Kacew

**Affiliations:** 1 Department of Medical Services and Techniques, Medical Laboratory Techniques Programme, Vocational School of Health Services, Antalya Bilim University, 07190 Antalya, Turkey; 2 F.M. Kirby Neurobiology Center, Boston Children’s Hospital, 300 Longwood Avenue, Boston, MA 02115, USA; 3 Department of Neurobiology, Harvard Medical School, Boston, MA 02115, USA; 4 Institute of Population Health, R. Samuel McLaughlin Centre for Population Health Risk Assessment, University of Ottawa, Ottawa, ON K1N 6N5, Canada

Humans and animals may be exposed on a continuous daily basis to a mixture of environmental contaminants that may act on several organ systems through differing mechanisms [1] resulting in adverse consequences. Environmental contamination now constitutes a major global issue with adverse effects on health of the ecosystem and food security. Globally, air pollution alone produces millions of premature deaths annually, predominantly associated with from lung cancer, chronic obstructive pulmonary disease (COPD), asthma, stroke, heart failure, and respiratory infections, according to the World Health Organization (WHO) [2]. It is noteworthy that 99% of humanity breathes air containing contaminants above recommended levels.

In order to mitigate contamination and diminish e our burden of pollutant -related diseases, we need to devise target-specific strategies to prevent or decrease exposure. To that end, risk assessment attributed to exposure to synthetic or and naturally occurring contaminants is necessary and; thus evidence obtained from toxicity studies appears to be of critical importance. Comprehensive efforts need to be undertaken to search for possible underlying mechanisms of action for each pollutant to establish toxic potential and safe limits through both in vitro and in vivo animal testing approaches. This issue focused on environmental pollutants including heavy metals, pesticides, nanoparticles, micro-nanoplastics, indoor air pollutants, pharmaceuticals, and industrial toxicants with effects on human health, risk assessment, and relationship between various diseases and environmental pollutants. Human exposure to environmental pollutants may initiate adverse effects including neurotoxicity, carcinogenicity, infertility, and metabolic disorders. Therefore, research into possible mechanisms of action for environmental contaminants is of critical importance for the well-being of humans and animals [3].

Over the last couple of decades, novel in vitro and in vivo methods and techniques were developed in the scientific discipline genotoxicology, enabling investigators to quantify genotoxicity attributed to exposure to certain compounds [4,5]. Acute or chronic exposure to environmental contaminants is known to be associated with several adverse health conditions, including cancer, impaired immune and reproductive function, as well as imbalanced gastrointestinal microbiota, which regulates a range of host metabolic and immune processes. The aims of this topic are to present a comprehensive overview of different studies carried out with in vivo and in vitro model organisms and the potential risk of environmental pollutants exposure to human health. In this Topic, 20 original articles, 6 reviews and 1 communication were collected, as presented in Table 1 with a particular focus on alcohol-based hand sanitizers, polycyclic aromatic hydrocarbons, monochromatic light pollution, paraben as an endocrine disruptors, heavy metal pollution attributed to antimony and arsenic of mines in the soil, water, and sediments, groundwater with high fluoride, virus transmission from heating, ventilation, and air conditioning systems of urban subways, chronic home radon exposure, organotin compounds, heavy metal pollutants including mercury, lead, cadmium, polypropylene microplastics, ventral body wall defects in chick embryos, microcystin-LR as an aquatic toxin, N-nitroso compounds, methylmercury as a global pollutant, triazine herbicides, persistent organic pollutants, bisphenol A and trace metals, autophagy, nano- and micro-sized polystyrene particles, tributyltin as an environmental contaminant, polybrominated diphenyl ethers, and per- and polyfluoralkyl substances. Most of the examined compounds originated from natural sources, whereas some semi-synthetic derivatives were also identified and discussed. The most recent findings on the effects of compounds and their constituents in treating various toxic outcomes and genotoxicity are discussed. These studies summarize our current knowledge based upon previous in vitro and in vivo research that scrutinized the influence of several environmental contaminants on various mammalian and non-target model organisms at several genetic, cellular, and molecular levels, as well as potential mechanisms underlying toxicity.

Table 1 schematically illustrates the content of this Topic, with all the contributions published in the five participating journals.

## Figures and Tables

**Table 1:** Original articles and reviews collected in the six journals participating in the Topic using different in vitro and in vivo model systems.

Title	Author	Journal	Year	DOI
Evaluation of the Safety and Efficacy of Hand Sanitizer Products Marketed to Children Available during the COVID-19 Pandemic	[6]	*IJERPH*	2022	https://doi.org/10.3390/ijerph192114424 (accessed on 1 February 2022)
Health Risk Assessment of Dermal Exposure to Polycyclic Aromatic Hydrocarbons from the Use of Infant Diapers	[7]	*IJERPH*	2022	https://doi.org/10.3390/ijerph192214760 (accessed on 1 February 2022)
Monochromatic Light Pollution Exacerbates High-Fat Diet-Induced Adipocytic Hypertrophy in Mice	[8]	*Cells*	2022	https://doi.org/10.3390/cells11233808 (accessed on 1 February 2022)
Impact of Paraben Exposure on Adiposity-Related Measures: An Updated Literature Review of Population-Based Studies	[9]	*IJERPH*	2022	https://doi.org/10.3390/ijerph192316268 (accessed on 1 February 2022)
Leaching Mechanism and Health Risk Assessment of As and Sb in Tailings of Typical Antimony Mines: A Case Study in Yunnan and Guizhou Province, Southwest China	[10]	*Toxics*	2022	https://doi.org/10.3390/toxics10120777 (accessed on 1 February 2022)
Relationship of Fluoride Concentration to Well Depth in an Alluvial Aquifer in a Semiarid Area	[11]	*Environments*	2022	https://doi.org/10.3390/environments9120155 (accessed on 1 February 2022)
Reducing Virus Transmission from Heating, Ventilation, and Air Conditioning Systems of Urban Subways	[12]	*Toxics*	2022	https://doi.org/10.3390/toxics10120796 (accessed on 1 February 2022)
Chronic Home Radon Exposure Is Associated with Higher Inflammatory Biomarker Concentrations in Children and Adolescents	[13]	*IJERPH*	2023	https://doi.org/10.3390/ijerph20010246 (accessed on 1 February 2022)
Organotin Antifouling Compounds and Sex-Steroid Nuclear Receptor Perturbation: Some Structural Insights	[14]	*Toxics*	2023	https://doi.org/10.3390/toxics11010025 (accessed on 1 February 2022)
Health Risk Assessment for Human Exposure to Heavy Metals via Food Consumption in Inhabitants of Middle Basin of the Atrato River in the Colombian Pacific	[15]	*IJERPH*	2023	https://doi.org/10.3390/ijerph20010435 (accessed on 1 February 2022)
Exposure to Polypropylene Microplastics via Oral Ingestion Induces Colonic Apoptosis and Intestinal Barrier Damage through Oxidative Stress and Inflammation in Mice	[16]	*Toxics*	2023	https://doi.org/10.3390/toxics11020127 (accessed on 1 February 2022)
Y-27632 Impairs Angiogenesis on Extra-Embryonic Vasculature in Post-Gastrulation Chick Embryos	[17]	*Toxics*	2023	https://doi.org/10.3390/toxics11020134 (accessed on 1 February 2022)
Downregulation of LncRNA GCLC-1 Promotes Microcystin-LR-Induced Malignant Transformation of Human Liver Cells by Regulating GCLC Expression	[18]	*Toxics*	2023	https://doi.org/10.3390/toxics11020162 (accessed on 1 February 2022)
Association of Dietary Nitrate, Nitrite, and N-Nitroso Compounds Intake and Gastrointestinal Cancers: A Systematic Review and Meta-Analysis	[19]	*Toxics*	2023	https://doi.org/10.3390/toxics11020190 (accessed on 1 February 2022)
Subchronic Low-Dose Methylmercury Exposure Accelerated Cerebral Telomere Shortening in Relevant with Declined Urinary aMT6s Level in Rats	[20]	*Toxics*	2023	https://doi.org/10.3390/toxics11020191 (accessed on 1 February 2022)
Triazine Herbicides Risk Management Strategies on Environmental and Human Health Aspects Using In-Silico Methods	[21]	*IJMS*	2023	https://doi.org/10.3390/ijms24065691 (accessed on 1 February 2022)
Development of an Improved Sulfur-Oxidizing Bacteria-Based Ecotoxicity Test for Simple and Rapid On-Site Application	[22]	*Toxics*	2023	https://doi.org/10.3390/toxics11040352 (accessed on 1 February 2022)
A Realistic Mixture of Persistent Organic Pollutants Affects Zebrafish Development, Behavior, and Specifically Eye Formation by Inhibiting the Condensin I Complex	[23]	*Toxics*	2023	https://doi.org/10.3390/toxics11040357 (accessed on 1 February 2022)
Protective Effects of Selenium Nanoparticles against Bisphenol A-Induced Toxicity in Porcine Intestinal Epithelial Cells	[24]	*IJMS*	2023	https://doi.org/10.3390/ijms24087242 (accessed on 1 February 2022)
*Drosophila* as a Robust Model System for Assessing Autophagy: A Review	[25]	*Toxics*	2023	https://doi.org/10.3390/toxics11080682 (accessed on 1 February 2022)
Uptake of Breathable Nano- and Micro-Sized Polystyrene Particles: Comparison of Virgin and Oxidised nPS/mPS in Human Alveolar Cells	[26]	*Toxics*	2023	https://doi.org/10.3390/toxics11080686 (accessed on 1 February 2022)
Environmental Health and Toxicology: Immunomodulation Promoted by Endocrine-Disrupting Chemical Tributyltin	[27]	*Toxics*	2023	https://doi.org/10.3390/toxics11080696 (accessed on 1 February 2022)
Toxic Effects and Mechanisms of Polybrominated Diphenyl Ethers	[28]	*IJMS*	2023	https://doi.org/10.3390/ijms241713487 (accessed on 1 February 2022)
Maternal Serum Concentrations of Per- and Polyfluoroalkyl Substances in Early Pregnancy and Small for Gestational Age in Southern Sweden	[29]	*Toxics*	2023	https://doi.org/10.3390/toxics11090750 (accessed on 1 February 2022)
Transfer of Bisphenol A and Trace Metals from Plastic Packaging to Mineral Water in Ouagadougou, Burkina Faso	[30]	*IJERPH*	2023	https://doi.org/10.3390/ijerph20206908 (accessed on 1 February 2022)
Environmental Endocrinology: Parabens Hazardous Effects on Hypothalamic–Pituitary–Thyroid Axis	[31]	*IJMS*	2023	https://doi.org/10.3390/ijms242015246 (accessed on 1 February 2022)
Mixture Effects of Bisphenol A and Its Structural Analogs on Estrogen Receptor Transcriptional Activation	[32]	*Toxics*	2023	https://doi.org/10.3390/toxics11120986 (accessed on 1 February 2022)
